# Verbal/Social Autopsy in Niger 2012–2013: A new tool for a better understanding of the neonatal and child mortality situation

**DOI:** 10.7189/jogh.06.010602

**Published:** 2016-06

**Authors:** Khaled Bensaïd, Asma Gali Yaroh, Henry D Kalter, Alain K Koffi, Agbessi Amouzou, Abdou Maina, Narjis Kazmi

**Affiliations:** 1 UNICEF, Niger country office, Niamey, Niger (retired); 2 Ministry of Health, Niamey, Niger; 3 Department of International Health, Johns Hopkins Bloomberg School of Public Health, Baltimore, MD, USA; 4 UNICEF, New York City, NY, USA; 5 Institute National des Statistics, Niamey, Niger

## Abstract

Niger, one of the poorest countries in the world, recently used for the first time the integrated verbal and social autopsy (VASA) tool to assess the biological causes and social and health system determinants of neonatal and child deaths. These notes summarize the Nigerien experience in the use of this new tool, the steps taken for high level engagement of the Niger government and stakeholders for the wide dissemination of the study results and their use to support policy development and maternal, neonatal and child health programming in the country. The experience in Niger reflects lessons learned by other developing countries in strengthening the use of data for evidence–based decision making, and highlights the need for the global health community to provide continued support to country data initiatives, including the collection, analysis, interpretation and utilization of high quality data for the development of targeted, highly effective interventions. In Niger, this is supporting the country’s progress toward achieving Millennium Development Goal 4. A follow–up VASA study is being planned and the tool is being integrated into the National Health Management Information System. VASA studies have now been completed or are under way in additional sub–Saharan African countries, in each through the same collaborative process used in Niger to bring together health policy makers, program planners and development partners.

There has been substantial global progress towards achieving the Millennium Development Goal 4 (MDG 4) of reducing child mortality by two–thirds between 1990 and 2015. However, few countries in Sub Saharan Africa will achieve MDG 4, and the regional under 5 mortality rate of 98 deaths per 1000 live births remains the highest in the world. Neonatal mortality has been the most difficult component to overcome, with the level in Sub Saharan Africa remaining at 32 deaths per 1000 live births [1]. Strategies required to further decrease child mortality include expanding health promotive and disease preventive practices in the community and by the health system, improved illness recognition and careseeking by child caregivers, and increased access to quality health care [2,3]; while for neonates, additional measures include the provision of quality antenatal care, skilled birth attendance and normal newborn care, and increased access to basic and comprehensive emergency obstetric and neonatal care [4-7].

The delivery of health services is guided by health policies and implemented through programs developed at the national or sub–national level. However, the information needed for evidence–based decision making is often lacking or of low quality and not widely accessible in developing countries [8,9]. Obtaining reliable, valid and timely data, especially data related to the causes and determinants of neonatal and child mortality is challenging. National health management information systems (HMIS) collect data on a regular basis and have the potential to support ongoing service improvement and decision–making. Yet, the quality of HMIS data in developing countries is poor; and it also may not be generalizable due to its collection in facilities with less than universal coverage [10,11]. The integrated verbal and social autopsy (VASA) tool and methods were developed to help overcome these limitations by supporting the collection of reliable and high quality population–based data on community– and health system–related maternal morbidity and neonatal and child mortality indicators.

A second critical factor in achieving positive results is the health policy and program planning process whereby study findings are utilized for decision–making. Case studies in Ethiopia highlighted that in addition to the lack of data, other major barriers to using data for decision making were awareness that data exists, accessibility and formatting of data, poor demand and lack of capacity of policy makers to appreciate and use data for informed decision making [12]. A study from Madagascar found that if data users and producers work together from the point of development of tools, the resulting data would be easy to understand and better used both by program implementers and policy makers [13]. As shown by work in Egypt [14] and Mexico [15], interest and commitment on the part of government to use the data to develop needed health programs also are key elements. This was also supported by the fact that the commitment of the government of Uganda to ensure data availability at district level and their willingness to support implementation of strategies improved women’s access to the selected health services [16].

Prior evidence supports that the use of verbal autopsy data in health policy development and program planning can lead to significant improvement in health outcomes. A verbal autopsy study in Kenya that focused on data use for decision making found that visual synthesis of data facilitated the use of information in health decision making at the district health system level and promoted program improvement [17]. In Egypt, making use of available verbal autopsy information in designing high mortality impact interventions resulted in a major decline in maternal mortality [14]; a similar pattern was observed in Indonesia [18]. Other countries also have utilized maternal and child mortality data to guide the development of interventions as well as in making informed policy and program decisions [15,19-21].

Niger is one of the few countries in sub–Saharan Africa that have achieved a tremendous reduction in mortality among children under the age of five years, placing the country on track to achieve the MDG 4. In 1990, over three in ten newborns in Niger were dying before reaching age five. The country has taken vigorous measures in the past decade to tackle this high mortality burden and succeeded in reducing child mortality by 65% between 1990 and 2012, with an annual rate of reduction of 4.8% [1]. Encouraged by this success, the Government has decided to strengthen its child survival strategy by addressing additional causes and determinants of mortality with a special emphasis on newborns, whose mortality rate declined insignificantly from 39 deaths per 1000 live births in 1998 to 33 in 2009 [22].

Based on the experiences of other countries in the use of mortality data for guiding program planning, and on the results of a recent study in Niger [22] that demonstrated the power of implementing “high mortality impact” interventions, the Government of Niger decided to conduct a VASA study. The study was implemented in 2012–2013 by the National Statistics Institute (INS) and a VASA working group including the Ministry of Health (MoH), the Niger country office of UNICEF and other partners in collaboration with the Johns Hopkins Bloomberg School of Public Health (JHSPH), which provided technical assistance on behalf of the WHO/UNICEF–supported Child Health Epidemiology Reference Group (CHERG).

The approach was to collect detailed information on the biological causes (verbal autopsy) and household, community and health system determinants (social autopsy) of death. The study was intended to provide critical data needed to revise the child survival strategy by reorganizing and reorienting health services and health interventions for improved care of pregnant and delivering mothers, newborns and children. Also critical to the goal of achieving maximal impact on maternal and child health policy and program development was the plan for the study partners to collaboratively analyze, interpret and disseminate the findings throughout the country.

## VASA METHODOLOGY

Mapping deaths identified by the 2010 Niger National Mortality Survey (NNMS) was used to select nationally representative samples of 605 neonatal deaths (0–27 days) and 605 infant and child deaths (1–59 months) that occurred between 2007 and 2010. The VASA study considers the biological causes of death and three levels of determinants, including family (cultural), community (social) and health system factors that affect access to and utilization of health promotive and disease preventive and curative interventions. The data collection tools include the Population Health Metrics Research Consortium verbal autopsy questionnaire [23] to determine the biological causes of death and the CHERG social autopsy questionnaire [24] to collect data on the determinants. The two questionnaires are chronologically blended together to identify the cause of death and actions that might have been taken before and during the illness to prevent the death.

The analytic methods are fully described elsewhere [25,26]. In brief, the causes of death were determined through the use of expert verbal autopsy algorithms arranged in a hierarchy (EAVA method) to select the primary cause of death, and by one physician certifying the VA underlying cause of death using pre–specified minimal diagnostic criteria together with her clinical judgment (PCVA method), followed by comparison of the EAVA and PCVA diagnoses to help assess their plausibility and reliability. The SA analysis examined the prevalence of preventive factors along the continuum of normal maternal, newborn and child care, and of potential curative factors and constraints to careseeking for the fatal illnesses of the neonates and children along the steps in the Pathway to Survival [24]. In addition, for neonatal deaths we examined maternal pregnancy and delivery complications, as well as careseeking, constraints to careseeking, and the care received for the complications.

It is important to note that conducting the VASA study on the platform of the earlier mortality survey greatly reduced the cost of the VASA compared to a conventional survey through direct interviewing of homes where deaths were reported by the NNMS.

The VASA study was approved by the National Consultative Ethics Committee of the Niger Ministry of Health and the Institutional Review Board of the Johns Hopkins Bloomberg School of Public Health. Informed consent was given by all study participants prior to their being interviewed.

## SOME EXAMPLES OF RESULTS

The VASA study provided information previously not available in Niger either at the local or national level. Of particular concern to the country was the need for data on the causes of neonatal and child deaths, maternal complications contributing to neonatal mortality, and some determinants of access to care such as problems faced by mothers to go to a health care facility for their maternal complications late in pregnancy and during labor and delivery and for their sick neonates and children.

The verbal autopsy showed that from 2007–2010 two thirds of neonatal deaths occurred during the first six days of life; more than one in two newborns died from infectious diseases and one in six succumbed to neonatal asphyxia. As for deaths of children aged 1–59 months, just under half occurred before the first birthday, and infectious diseases caused more than 90% of the deaths.

Unexpected results were revealed. For example, in a country where two out of three births take place at home, the pregnant woman herself decided the place of birth in seven of 10 cases, against 18% for her husband and 6% for her mother and mother–in–law.

Paradoxes were identified that require further study to better understand certain behaviors. While five in seven women delivered at home, less than one in three of these women said they had a problem that prevented them from giving birth at a health care provider. Problems of distance and transport were cited most often, by almost 80% of these women, as an obstacle to delivering at formal services, against only 4% of the women who were afraid to be exposed to a male health provider.

In addition, nearly 96% of the mothers and other caregivers of the neonates whose illness began at home reported a possibly severe or severe illness sign or symptom such as fast breathing, fever, or not able to feed, but many either did not recognize or minimized their meaning and importance. As a result, two–thirds of the mothers did not deem it necessary to seek care outside of the home even in the presence of these illness signs. For those who did seek formal health care, the average time before deciding to go was nearly 2 days.

Finally, the study showed very limited access to hospital services, which were received, respectively, by only 3.1% of newborns delivered at home and 13.3% of children aged 1–59 months. Referral was minimal for the vast majority who were seen by a first level provider. Only 8.7% of 69 neonates and 19.0% of 306 children who left the first provider alive were referred.

The complete study results for neonates and children 1-59 months are available in a prior publication [25] and two new papers [27,28] in the current issue of the Journal.

## USE OF THE VASA DATA FOR POLICY AND PROGRAM DEVELOPMENT

A favorable environment for effective use of the VASA data existed in Niger from the start due to the government's interest in the study as a means of continuing the momentum toward decreasing child mortality and achieving MDG 4. This was reinforced by the CHERG’s emphasis on the early formation of a country working group (CWG) of child survival experts, policy makers and program planners and their active participation in the analysis, interpretation and dissemination of the study findings to a wide circle of stakeholders.

An early meeting of CHERG, the INS and the MoH identified the crucial need for information on the causes and determinants of under–five deaths to help guide the review of the 2011–2015 National Health Development and Child Survival plan and the National Maternal and Neonatal roadmap. The first CWG meeting in 2012 included participants from the INS, MoH, UNICEF and WHO.

Two dissemination meetings were held in 2013. The first brought together regional and central health authorities of Niger and neighboring countries to get acquainted with the VASA data, of a type rarely obtained before and on a national scale, and to broadly assess what the tool itself can produce as strategic information for the planning and evaluation of health interventions. The second involved 42 district health teams, which developed health policy and program recommendations and considered operational aspects of including the VASA in a new pillar of the National HMIS.

## RECOMMENDATIONS AND THEIR IMPLEMENTATION

Three sets of recommendations emerged from these meetings. The initial assessment of the implementation of recommendations [29] was quite encouraging, especially as it was conducted only six months after the last dissemination workshop held in November 2013.

1. The first set of recommendations concerned strengthening the existing maternal and child health programs or interventions in light of the VASA study findings. Implementation progressed as follows

i. The VASA study found that less than one–third of women with a neonatal death delivered with skilled attendance. As a result, it was recommended that the existing policy of free care for children be extended to deliveries. UNFPA had already funded free deliveries on a pilot basis in four of the country’s eight regions in 2010, and following an evaluation extended this from 2011 to 2014. Based on the VASA meeting recommendation, the concept note on the reduction of maternal and neonatal mortality currently being drafted by the MoH is to include national scaling of the program of free deliveries. From 2015, funding is being provided by the Reproductive Maternal and Newborn Child Health (RMNCH) initiative through UNFPA.

ii. In response to the VASA finding that nearly 80% of the women with a problem in reaching a health facility for delivery cited lack of transportation and distance as the main barriers, the Government acquired 150 motorcycle ambulances for the transport of pregnant women referred from first level health facilities to hospital and is negotiating with mobile phone companies to improve communications between these facilities for the referral of women in labor. For transportation problems from home to a facility the government is also pilot testing bovine or mule wagons and boats in island areas.

iii. The VASA study finding that most neonates who died were not taken outside the home for health care provided an opportunity to discuss the need to accelerate taking community newborn case management to scale. The MoH held a workshop in early November 2014 to discuss the initial experience of scaling up mother and newborn community case management to 45 health posts in three health districts. Extension to 208 health posts in 20 additional health districts is planned, with a training of trainers’ workshop having been conducted in May 2015.

iv. The VASA finding of limited access to quality health care led to accelerating the recruitment of qualified nurses for health posts and their transformation into health centers from an average of 50 health posts transformed per year before 2014 to nearly 100 units in 2014.

v. While introduction of the Pneumococcal vaccine was already planned, this was reinforced by the VASA findings, which showed that pneumonia was one of the main causes of death of under–5 children.

vi. The VASA study found that 96% of caregivers were able to report their deceased child’s signs of severe illness, yet 69% and 21%, respectively, of newborns’ and children’s caregivers did not seek health care for the illness. A recommendation was made to develop an integrated communications plan on danger signs in pregnancy and child illness, but as of yet significant progress has not been made. The first step is to articulate a global view of communication and a comprehensive communication plan that can be negotiated with partners for its implementation.

2. The second set of recommendations concerned the use of the VASA findings in the planning and evaluation of district annual action plans and revision of the maternal and neonatal mortality reduction roadmap, the child survival strategy and the national health development plan. Implementation of the recommendations progressed as follows:

i. Regarding the maternal and neonatal mortality reduction roadmap:

a. A working group was established to write the roadmap concept note, establish consensus and the means of fundraising.

b. The VASA findings that antepartum and intrapartum hemorrhage, maternal sepsis and eclampsia were the three main maternal complications contributing to neonatal deaths raised the awareness of decision makers and led to the implementation of new case management interventions. A census of existing human resources and technical equipment was conducted, followed by the endowment of essential equipment to all 42 districts. A program for capacity building in case management of the three complications was conducted and the interventions have been implemented.

c. Based on the VASA study finding that a very low percentage of the neonates that died were delivered by Caesarean Section, the program for training general practitioners in the “surgery of the district” was accelerated, including abdominal emergency procedures such as Caesarian Section.

ii. Revision of the child survival strategy involved the full utilization of the VASA data:

A ministerial decree on the mission of the national committee for the revision of the strategy document was signed [30], UNICEF mobilized the necessary funding, a National Technical Working Group was officially installed and its terms of reference were finalized. The final revision was entered in the MoH 2014 work plan and the UNICEF annual work plan.

The review coincided with the scaling up of the Reproductive, Maternal, Newborn and Child Health H4+ initiative involving four UN agencies (UNFPA, World Bank, UNICEF, WHO) and other partners and donors, which provided a portion of the necessary funds for the review. Many countries including Niger will benefit from this activity. A concept note was developed based on the revised child survival strategy with the goal of sharing the new strategy for its adoption and funding by all partners and stakeholders to further accelerate the reduction of child mortality.

iii. Regarding the national health development plan, a working group was formed to review the 2010–2015 plan and elaborate the 2016–2020 plan on the basis of available evidence, including the next VASA study.

3. The third set of recommendations concerned strengthening the National HMIS through integration of the VASA tool with the UNICEF Multiple Indicator Cluster Survey (MICS) or the Niger National Mortality Survey. Implementation of the recommendations progressed as follows:

i. The VASA tool was officially adopted by the political leadership, and discussions with the National Institute of Statistics to formalize its integration in the National System for Development of Statistics (NSDS) are under way. This will be accompanied by new regulatory measures to define the roles and responsibilities of the involved parties. Also, the process for adopting and integrating the VASA tool within the National HMIS is being identified.

ii. Possible means of conducting the VASA on a regular basis are being examined, including a combined MICS/VASA survey and integration with all future Niger National Mortality Surveys. Technical assistance may be required for the initial round, both for integration of the VASA with the survey and its articulation with the entire HMIS. A possible obstacle concerns the availability of funding, though some partners, including the World Bank and the Common Fund/Swap partners have already shown interest.

## DISCUSSION

Like many countries in the region, the formulation of health policies and strategies in Niger is based on analyses of surveys that are conducted every five or six years, such as the MICS and Demographic and Health Survey. These surveys provide information mostly on system performance in terms of coverage of preventive and curative services, but rarely on aspects related to population demand. The country’s routine health information system, which is the mainstay for the daily management of services and programs, deals almost exclusively with the surveillance of notifiable diseases. This pillar also suffers from limited reliability due to uncertainty of the relevant denominators, and so is rarely used for central planning of medium and long term programs. Lastly, some additional studies are occasionally conducted in limited geographic areas and used to analyze the situation of particular programs such as Reproductive Health. Faced with this ongoing gap in required data, as well as the evaluation of the 2010–2015 National Health Development Plan, achieving MDG 4 and the accelerated drive to reduce maternal mortality, reform of the HMIS has been an active topic of recent discussion. This has included a review of all indicators, the frequency of their production, and how they are used for better management of services and resources.

While recent years have seen an improvement in the coordination between departments and programs, difficulties remain in the use of data in the decision–making process. These include, among others, limited dissemination and use of data, often due to the organization of surveys and studies by individual programs leading to weak communication between programs and between programs and sources of support; failure to bring together all relevant departments overseeing programs and resources in the discussion of the data and planning exercises; inadequate financing resulting in weak field monitoring that fails to ensure the necessary feedback to decision makers; and, finally, in the type of data available, usually concerning the performance of intervention coverage and rarely identifying the demand for services or the barriers to access to care.

Several of the above problems evoke lessons learned in other developing countries regarding how to increase the utilization of data for health policy and program development, from the need for population–based, representative data [10] and the lack of data accessibility and low capacity of policy makers to use data for decision making in Ethiopia [12] to the need for better communication between data producers and users in Madagascar [13]. However, the situation in Niger additionally suggests the need for international donors and partners to not only support large surveys of interest to the global community, but also to more closely examine countries’ data needs and contribute to strengthening the processes that will promote the local use of data for improved policy and program development.

In 2007 Niger introduced an effort to use new determinants in the National HMIS, based on the model of Tanahashi [31] and mainly to identify key bottlenecks for preventive services, such as those used to calculate the availability of services and resources, access to services, utilization, adequate coverage and, finally, effective coverage. However, this endeavor was not supported by adequate data, nor by official technical guidelines or training documents for taking the effort to scale.

Thus, the need for population–based data on the determinants of access to care and barriers to the use of services as well as data on causes of death became imperative to accelerate the achievement of MDG 4. The VASA study helped fulfill these data needs, and its implementation model of bringing together a country working group of health policy makers, program planners and international partners to help analyze and interpret the findings, draw conclusions and recommendations and plan next steps, increased the likelihood of the information being used for the improvement of maternal and child health interventions.

The dissemination of the VASA results with the participation of all district health teams further responded to the need for the use of relevant, population–based data in a planning exercise bringing together decision makers, program managers and resource providers, and opened new horizons toward strengthening the health information system in particular and the system of planning, monitoring and evaluation for better governance in general. The VASA study corroborated past evidence of the benefits of utilizing verbal autopsy data for health policy development and program planning [16,17], like some past studies included the collection of extensive data on social determinants in addition to causes of death [19-21], and advanced on this past work through its highly collaborative implementation model.

The assessment exercise undertaken in mid–2014 [29] to examine the follow–up to the recommendations made at the second VASA dissemination meeting in November, 2013 helped revitalize the process. Contacts with the Minister of Health, assembling of the MoH team with INS staff, and providing feedback to the Technical and Financial Partners in the presence of the MoH team facilitated the taking of important decisions. It is apparent that, although the VASA study uniquely met the self–identified data needs of the Government of Niger and was undertaken through a highly collaborative process aimed at promoting the use of the data for evidence–based decision making, continued support from technical and resource partners is still critical to achieve this goal along the road to MDG 4.

## CONCLUSIONS

The VASA study allowed the health authorities in Niger to better understand the causes of death and constraints to accessing and utilizing care, as well as weaknesses of the health system in ensuring optimal responses to the health problems of mothers and children. The Niger Government and its health partners will continue their collaboration towards the goal of making the VASA tool a component of the HMIS and a basic reference of choice for developing strategic maternal and child health policies and programs. The experience of the VASA study in Niger helped to refine the tool, which has since been implemented in several additional countries.

Despite the critical mass of recommendations already implemented, continued input by technical and financial partners is still needed to stimulate the full utilization and integration of the VASA tool into the HMIS and the NSDS and monitoring of the development and implementation of the various recommendations. This will accelerate the process including identifying and supporting new funding.
